# Partnership Working in Health and Social Care: 2nd Edition 2014, Jon Glasby and Helen Dickinson, Publisher: Policy Press

**DOI:** 10.5334/ijic.4722

**Published:** 2019-07-04

**Authors:** Nereide Alhena Curreri

**Affiliations:** 1 University of Stirling, Ageing and Dementia, UK

**Keywords:** integrated care, integration, partnership working, organizational theory, joint working, collaboration

This book sets out to describe the development and implementation of integrated care and to debunk integrated care suppositions by examples from policy and research. It is authored by Jon Glasby and Helen Dickinson, editors of the series Better Partnership Working. In five chapters, the main characteristics of integrated care, from definitions and its effectiveness, to evaluation tools, are presented explicitly. The topics are discussed specifically in the UK context and the term ‘partnership working’ is used consistently, as an alternative for integrated care. The book makes a clear contribution to integrated care by dissecting what policy intends it to be, what practice demonstrates it to be (or not to be), and offers different frameworks to implement it. Boxes and tables are provided in each chapter in addition to examples from the literature. At the end of each chapter the authors offer reflective exercises, further readings to delve deeper into the topic, and other resources such as websites of associations and professional bodies either referenced in or relevant to the chapter.

The book begins with an informative preface stating that the second edition is a response to the more recent use of vocabulary, such as integration, joint working, person centred care, and partnerships, in policy. Additionally, in the preface, partnership working and integrated care are placed in the real life context of consumers and frontline professionals, and the UK policy context is explored.

Chapter 1 sets the foundation on ‘What is partnership working and why does it matter’. A variety of definitions are provided for key terms such as ‘partnership working’ and ‘integrated care’, demonstrating the lack of consensus and widespread use. The authors underline that whichever definition is used, it is vital for all key partners to share the same understanding and meaning. In addition, they point out that the term ‘partnership’ denotes three different concepts used to organize services: markets, hierarchies and networks. These are concisely described, and the next section illustrates how in reality policies have been cyclical rather than linear, changing from hierarchy-based health systems to market based, to network based, and back again.

Chapter 2 considers the evidence that partnerships are effective. Outcomes are shown to be regularly ignored in partnership studies through four different literature reviews and local examples, in which processes and structure tend to be the focus. The authors argue that descriptions of ideal situational conditions for partnerships are prevalent in the literature yet the ‘how to manage’ is not explored. Two useful frameworks, that possibly oversimplify reality according to the authors, are provided as partnership evaluation tools for practitioners and/or stakeholders. Furthermore, in this chapter, Glasby and Dickinson highlight the growing evidence that structural changes are not directly correlated with better outcomes for service users, or more functional partnerships. The authors suggest research make a shift in focus onto what does *not* work, as evidence for practice.

Chapter 3 introduces ‘hot topics’ in partnership working such as forced partnerships, accountability, responsibilities, focus on service users and the divide between health and social care. Forced partnerships are suggested to be a method of creating change in services by changing people’s behaviours via ideas and structural modifications. How to achieve clear accountability parameters in finances and duties remains vague and underdeveloped in most partnerships. A balance in governance arrangements is recommended to avoid the extremes of either delegating without trusting a partner or delegating to the point of disengaging from any responsibility. The question of whether partnerships are ‘good’ for service users is posed via an example illustrating that more power for providers and reduced choice are possible direct outcomes of partnerships. To assist partnerships in maintaining transparency and overall good practice, a comprehensive governance assessment tool is provided. The authors conclude that clinical and social practitioners are unconnected and interdisciplinary collaboration is rare due to policy contexts and differing priorities.

In Chapter 4, useful development frameworks and concepts are offered. The importance of focusing on outcomes is emphasized via the context-process-outcomes approach, which asks frontline services and practitioners to discuss: outcomes, what they want to achieve; context, how well do current services meet those outcomes; and process, what structures need to be developed to achieve those outcomes. The focus shifts from structures to outcomes, specifically outcomes that involve users. Next, each step of the approach is explored through definitions of types of outcomes and optimistic, pessimistic and realist approaches to partnership working. The authors argue that once partners are clear on the outcomes and the context, the ‘what to do next’ is the most complicated step. Key frameworks are provided to assist in designing outcome centred partnerships that explore laws of integration, depth versus breadth, modes of governance, approaches to leadership and levels of engagement. The book concludes with Chapter 5, where a concise list of practical recommendations for policy makers and local organizations and/or frontline services.

Overall, in a little less than 100 pages this book provides the reader a foundation on how integrated care is idealized and how it has been implemented. It examines the various themes of integrated care, or partnership working, such as structures, policy, context, people, needs and interests. It also illustrates how the process of integrating care is deemed more important than the outcomes. The authors expose the assumptions held about health and social services collaborating, about the positive impacts of integrated care and partnership working, and more specifically assumptions that there are consensus definitions for both.

Definitions and uses of key terms are critically considered to the point of leaving the reader uncertain of which are accurate. Including international examples and reports would enrich the text and provide context comprehensiveness.

The key take home message is that partnership working or integrated care is a means to an end, specifically outcomes, not an end to itself. The book is ideal for early career researchers and professionals as well as senior academics and practitioners wanting a concise, comprehensive overview of integrated care.

